# A new purification method for enhancing the immunogenicity of heat shock protein 70-peptide complexes

**DOI:** 10.3892/or.2012.2051

**Published:** 2012-09-21

**Authors:** YANWEI GAO, XIA CHEN, WEISHI GAO, YONG YANG, HULIN MA, XINJUN REN

**Affiliations:** 1 Department of Oncology, Inner Mongolia People’s Hospital, Hohhot 010017, Inner Mongolia; 2 Inner Mongolia Red Cross Blood Center, Hohhot 010017, Inner Mongolia; 3 Tianjin Medical University Eye Center, Tianjin 300384, P.R. China

**Keywords:** heat shock protein 70-peptide complexes, HER-2/neu oncogene protein, membrane protein, dendritic cells, CD8^+^ T cells, immunotherapy, cancer

## Abstract

When purified from a tumor, certain heat shock protein 70 (HSP70)-peptide complexes (PCs) can function as effective vaccines against the tumor from which the complexes were isolated. The immunogenic mechanisms of HSP70 preparations imply that tumor-derived HSP70-PCs exhibit antigens associated with antigen-presenting cells such as dendritic cells (DCs), inducing antigen-specific cytotoxic CD8^+^ T cells. However, some important membrane-resident tumor-associated peptides, such as the HER-2/neu (c-erbB2) oncogenic protein, cannot be purified from HSP70 by traditional methods. In the present study, a new approach for the purification of HSP70-PCs from HER-2-overexpressing breast cancer cells was established. The detergent 3-[(3-cholamidopropyl)dimethylammonio]-1-propanesulfonate (CHAPS) was used to obtain more effectual tumor peptides. The new purified product was named HSP70-HER-2-PC, and its immunological activities were determined. Traditionally purified HSP70-PCs (without CHAPS) and recombinant human HSP70-HER-2 protein complexes (recombined *in vitro*) were used as controls. These three HSP70-associated tumor antigenic complex pulsed dendritic cells (DCs) were used to stimulate an antitumor response. The mature DCs pulsed with HSP70-HER-2-PCs stimulated autologous T cells to secrete higher levels of type I cytokine compared to the two control groups. Moreover, DCs pulsed with HSP70-HER-2-PCs induced the most specific CD8^+^ T cells that specifically killed the same tumor cells. These findings provide a basis for new approaches in enhancing HSP70-based immunotherapy for HER-2-associated or other membrane antigenic peptide-related cancers.

## Introduction

Heat shock proteins (HSPs) are important intracellular molecular chaperones that play essential roles in the regulation of protein folding and translocation (1). They are specifically induced in response to various stress conditions such as heat, anoxia, and certain chemicals. HSPs can act as cytoprotective agents by binding to misfolded proteins, thereby protecting them from denaturation under cellular stress (2,3).

HSPs are overexpressed in many tumors and act at the crossroads of key intracellular processes in their role as molecular chaperones. HSPs associate with a vast array of tumor antigenic peptides and are implicated as chaperones in the formation of immunogenic complexes called HSP-peptide complexes (HSP-PCs) (4–6). Immunization with HSP-PCs purified from cancer cells provides protection against tumors derived from the same cancer cells from which the complexes were purified. The immunogenicity of HSP preparations is attributed to the antigenic peptides that they chaperone (7–11). Studies on the immunogenic mechanisms of HSP preparations have shown that tumor-derived HSP-PCs exhibit antigens associated with antigen-presenting cells such as DCs. Consequently, antigen-specific cytotoxic CD8^+^ T cells are induced (1,12). Hence, HSP-PCs purified from tumor cells possess the qualities of tumor vaccines. Numerous reports have confirmed HSP70 as one of the best choices for HSP-based tumor vaccine preparations (13,14).

Recent research on HSP70-based tumor immunotherapy is focused on how to obtain more effective and powerful peptides by the purification of HSP70-PCs from tumor cells. Membrane proteins containing many important tumor antigens, such as HER-2 protein, cannot be obtained from cancer cells through HSP70 using previous purification methods. Traditional methods cannot completely separate the cellular membrane, resulting in the loss of membrane-associated HSP70-PCs in the purified product. This limitation attenuates the antitumor immunological activities of purified HSP70-PCs.

A preliminary experiment by our group revealed that HER-2 protein and HSP70 are both highly expressed in the human breast cancer cell line SKBR-3. A co-immunoprecipitation experiment also indicated that HER-2 protein and HSP70 are bound in the tumor cells. The traditional method, combining hypotonic buffer with column chromatography (using ConA-Sepharose and ADP-agarose), was utilized to purify the HSP70-PCs from SKBR-3 cells. However, no HER-2 protein was found by sodium dodecyl sulfate polyacrylamide gel electrophoresis (SDS-PAGE) and immunodot analysis in the final product (data not provided).

In the current study, an improved HSP70-associated vaccine was produced based on a purification process involving the detergent 3-[(3-cholamidopropyl)dimethylammonio]-1-propanesulfonate (CHAPS). HER-2 protein was used to represent the membrane tumor antigens. HER-2 proteins with HSP70-PCs were obtained from the HER-2-overexpressed human breast cancer cell line SKBR-3. The new product was temporarily named HSP70-HER-2-PCs. Their immunological activities were determined by pulsing DCs and inducing specific CD8^+^ T cells. Traditionally purified HSP70-PCs and the recombinant human HSP70-HER-2 protein complex were used as controls.

## Materials and methods

### Culture of the cell line

Human breast cancer cell line SKBR-3 was purchased from Peking Union Medical College. Cells were cultured in RPMI-1640 medium (Gibco, Invitrogen Co., Carlsbad, CA, USA) supplemented with 10% heat-inactivated fetal calf serum (FCS) (HyClone, Logan, UT, USA), 2 mol/l L-glutamine (Gibco, Invitrogen Co.), 100 U/ml penicillin G and 100 μg/ml streptomycin at 37°C in a humidified atmosphere of 5% CO_2_.

### Purification of HSP70-HER-2-PCs

SKBR-3 cells were heated in a water bath at 42°C for 12 h followed by recovery for 2 h at 37°C in an atmosphere containing 5% CO_2_. After the treatment of heat shock, SKBR-3 cells were digested by 0.02% trypsin and then 1×10^8^ cells were homogenized in hypotonic buffer with CHAPS (Sigma Chemical Co., St. Louis, MO, USA) (50 mM Tris-Hcl, 150 mM NaCl, 1 mM phenylmethylsulfonyl fluoride, 1 mM sodium fluoride, and 5 mM CHAPS, pH 7.2) for 15 min at 0°C. After ultrasonication at 0°C for 30 min, the homogenate was centrifuged at 14,000 rpm at 4°C for 90 min. The supernatant was dialyzed against buffer A (20 mM Tris-Hcl, 150 mM NaCl, 1 mM CaCl_2_, 1 mM MnCl_2_, 0.5 mM phenylmethylsulfonyl fluoride, and 15 mM β-mercaptoethanol, pH 7.4) overnight at 4°C. The sample was then loaded onto an ConA-Sepharose column (Sigma Chemical Co.). Fluid was collected at a flow rate of 12 ml/h, that consisted of the ConA-Sepharose-unbound protein. The fraction was dialyzed against buffer B (20 mM Tris-HCl, 20 mM NaCl, 3 mM MgCl_2_, 1 mM MnCl_2_, 0.5 mM phenylmethylsulfonyl fluoride and 15 mM β-mercaptoethanol, pH 7.4) overnight at 4°C. The sample was applied to an ADP-agarose column (Sigma Chemical Co.) equilibrated previously with buffer B at a flow rate of 12 ml/h. The column was eluted by buffer B and buffer B containing 0.5 M NaCl until the protein was not detected by the Bradford method. The target protein was eluted by buffer B containing 3 mM ADP (Sigma Chemical Co.). The endotoxin level in the preparations was determined by Limulus amebocyte lysate (LAL) assay (Ocean Biologicals Co., China).

### Identification of HSP70-HER-2-PCs

The target protein obtained by purification was run on SDS-PAGE and detected by Coomassie Brilliant Blue staining. In an immunodot analysis, several harvested parts were separately dotted on the nitrocellulose membrane and dried at 37°C for 30 min. The membrane was blocked by TBST (20 mM Tris-HCl, 150 mM NaCl and 0.05% Tween-20, pH 7.4) as well as 5% (w/v) skimmed milk powder at room temperature for 1 h, and then incubated with mouse anti-human monoclonal antibody against HSP70 (Santa Cruz Biotechnology, Inc., USA) at a 1:500 dilution in TBST/milk at 4°C for 1 h. The membrane was thrice washed in TBST, incubated with horseradish peroxidase (HRP)-conjugated goat anti-mouse IgG (Santa Cruz Biotechnology, Inc.) at a 1:10,000 dilution in TBST/milk at 37°C for 1 h, and again thrice washed in TBST. Finally, the membrane was incubated with a chemiluminescence reagent (Santa Cruz Biotechnology, Inc.) for 1 min, and then exposed to Kodak autoradiography film for 20 sec.

Using the above described method, rabbit anti-human monoclonal antibody against HER-2 and fluorescein isothiocyanate (FITC)-conjugated goat anti-rabbit IgG (both from Santa Cruz Biotechnology, Inc.) were used to detect the HER-2 protein of the complex. The contents of HER-2 protein in the purified products were quantified by HER-2 ELISA kits (R&D Systems) according to the manufacturer’s instructions.

### Recombinant human HSP70-HER-2 protein complex prepared in vitro

Studies have shown that recombinant HSP protein complexes *in vitro* are able to strengthen the cytotoxic response of T cells against tumor cells (15). In this step, we produced the recombinant human HSP70-HER-2 protein complex for the control for the following research. The recombinant human HSP70 and the recombinant human HER-2 protein were both purchased from R&D Systems. The recombinant HSP70-HER-2 protein complex was generated by incubation of the recombinant human HSP70 and the recombinant human HER-2 protein in a 1:1 molar ratio at 43°C for 30 min and then at 37°C for 1 h. The binding was evaluated by co-immunoprecipitation and western bolt analysis. Briefly, the HSP70-HER-2 protein complex was incubated with rabbit anti-human monoclonal antibody against HER-2 (1:100) at room temperature for 2 h. The immune complex was then precipitated by incubation with protein A-Sepharose CL-4B (20 μl/ml; Amersham Pharmacia Biotech AB, Uppsala, Sweden) and rotating for 8 h on ice. Samples were then washed eight times with washing buffer (1 M Tris-HCl, 5 M NaCl, 0.5 M EDTA and 0.1% Triton X-100, pH 7.4) at 4°C to remove any nonspecific binding of the recombinant proteins to protein A-Sepharose. The beads were then added with 2X SDS sample buffer, boiled for 5 min, and subjected to SDS-PAGE, followed by probing with the mouse anti-human monoclonal antibody against HSP70 (1:100) at room temperature for 1 h. Western blot analysis was performed using HRP-conjugated goat anti-mouse IgG (1:10,000) and FITC-conjugated goat anti-rabbit IgG (1:10,000). During analysis, the nitrocellulose membrane was incubated for 1 min with a chemiluminescence reagent followed by exposure to Kodak autoradiography film for 20 sec.

### Preparation of DCs and CD8^+^ T cells

DCs were generated as described with some modifications (16). Briefly, peripheral blood mononuclear cells (PBMCs) were isolated from heparinized venous blood of healthy donors (from Inner Mongolia Red Cross Blood Center, China) by Ficoll-Hypaque (1.077 g, Bei Jing Zhong Shan Jin Qiao Co., China) density gradient centrifugation and cultured in RPMI-1640 medium containing 10% FCS for 2 h. The non-adherent cells were collected for generating CD8^+^ T cells and the adherent cells were cultured for 7 days in RPMI-1640 medium containing 10% FCS, 800 U/ml recombinant human granulocyte-macrophage colony stimulating factor (GM-CSF) and 500 U/ml recombinant human interleukin-4 (IL-4) (both from R&D Systems, Inc., USA) for generating DCs. The media along with the necessary cytokines were half-refreshed every other day and 50 U/ml tumor necrosis factor-α (TNF-α) (R&D Systems, Inc.) was added to the culture medium on the sixth day.

CD8^+^ T cells were harvested from the non-adherent fraction. Briefly, non-adherent cells were resuspended in RPMI-1640 medium containing 10% FCS, 100 U/ml penicillin G, and 100 μg/ml streptomycin. Recombinant human interferon (IFN)-γ (1,000 IU/ml) (PeproTech Inc., USA) was added on day 0. After 24 h of incubation, 50 ng/ml of mouse anti-human monoclonal antibody against CD3 (Becton, Dickinson and Co., BD, USA), 100 U/ml recombinant human interleukin (IL)-1β (R&D Systems, Inc.) and 300 U/ml recombinant IL-2 (PeproTech Inc., USA) were added. Cells were incubated at 37°C in a humidified atmosphere of 5% CO_2_ and subcultured every third day in fresh complete medium with 300 U/ml IL-2 at 2×10^6^ cells/ml.

### Fluorescence immunostaining

DCs (1×10^5^) were pulsed with 10 μg of the HSP70-HER-2-PCs purified from SKBR-3 at 37°C for 12 h. After washing with phosphate-buffered saline (PBS), fixation in 4% paraformaldehyde, and permeabilization with 0.5% Triton X-100, the cells were blocked in 3% bovine serum albumin (BSA) at 4°C for 1 h. The cells were then incubated with mouse anti-human monoclonal antibody against HSP70 and rabbit anti-human monoclonal antibody against HER-2 at a 1:100 dilution in PBS at 37°C for 1 h, and then thrice washed in PBS. Rhodamine-conjugated goat anti-mouse IgG (Santa Cruz Biotechnology, Inc.) and FITC-conjugated goat anti-rabbit IgG were added at 1:50 dilution in PBS at 37°C for 30 min in the dark. The cells were again thrice washed in PBS and smeared on the slides. Images were acquired using an Olympus DP71 microscope and analyzed using basic software (Olympus).

### ELISPOT assay

An ELISPOT assay was performed to assess the IFN-γ production of autologous T cells using an IFN-γ ELISPOT kit (R&D Systems, Inc.). DCs were divided into four groups of 1×10^5^ cells each, and pulsed by different ways for 12 h. Group A received GM-CSF and IL-4 only, group B received 10 μg of HSP70-HER-2-PCs purified from SKBR-3, group C received 10 μg of the HSP70-PCs purified from SKBR-3 by a method without CHAPS, and group D received 10 μg of recombinant human HSP70-HER-2 protein complex. After washing with PBS, the four groups of DCs were cocultured with autologous T cells isolated by a nylon wool column at a 1:10 ratio in a 96-well culture plate (NUNC, Roskilde, Denmark) in the presence of 20 U/ml IL-2 for 7 days, respectively. The stimulated T cells (1×10^4^/well) as effector cells and SKBR-3 cells (5×10^3^/well) as target cells were transferred to the ELISPOT plate and incubated at 37°C for 18 h. The level of IFN-γ was detected as described in the IFN-γ ELISPOT kit manual with an automated ELISPOT reader system (Biosys Co., Germany).

### Induction of specific CD8^+^ T cells by DCs pulsed with HSP70-HER-2-PCs and in vitro cytotoxicity test

Specific cytolytic activities of CD8^+^ T cells were determined by an LDH release assay. Autologous CD8^+^ T cells were obtained by using the method described above. Cells were divided into four groups of 1×10^6^ cells each. Then four groups of CD8^+^ T cells were cocultured with four groups of autologous DCs which was pulsed by different ways (mentioned above) at a 10:1 ratio respectively. All the mixture was treated with 300 U/ml IL-2 in a 96-well plate for one week and named group A, B, C and D, correspondingly. After one week of coculture, four groups of CD8^+^ T cells were used as effector cells in the assay using LDH cytotoxicity detection kit (BioVision Inc., USA). SKBR-3 cells were used as target cells in the assay. Briefly, target cells and effector cells were resuspended in assay medium (RPMI-1640 with 1% BSA), and then target cells (1×10^4^ cells/well) were cocultured with effector cells at different ratios (1:5, 1:10, and 1:20) in a 96-well round-bottomed culture plate at 37°C. After incubation for 4 h, cells were centrifuged at 250 rpm for 10 min and the cell-free supernatant was collected and transferred to another ELISA plate for LDH assay. LDH detection mixture (100 μl/well) was then added and incubated in the dark for 30 min at room temperature. After adding 50 μl stop solution per well, the absorbance of the samples was measured by ELISA reader at 490 nm as reference wavelength. The spontaneous release of LDH by target cells or effector cells was assessed by incubation of target cells in the absence of effector cells and vice versa. The maximum release of LDH was determined by incubation of target cells in 1% Triton X-100 in assay medium. The percentage of specific cell-mediated cytotoxicity was determined by the following formula: Cytotoxicity (%) = [(effector and target mixture - effector spontaneous - target spontaneous)/(maximum - target spontaneous)] ×100.

### Generation of HER-2-knockdown cells

HER-2 small interfering (si)RNA (human HER-2 siRNA, Santa Cruz Biotechnology, Inc.) was added at a final concentration of 30 nM to knock down the expression of HER-2 in SKBR-3 cells. Lipofectamine 2000 (Invitrogen Co., Carlsbad, CA, USA) was used to transfect the siRNA into cells according to the instructions of the manufacturer. Western blot analysis was performed, as described previously, to investigate the ability of siRNA to suppress HER-2 expression. β-actin was used as the internal control.

### HER-2 specificity detection

The anti-tumor activities of the HSP70-HER-2-PCs were further detected against MDA-MB-453 (HER-2 overexpression) and HER-2-knockdown SKBR-3 cells to demonstrate the HER-2 specificity of HSP70-HER-2-PCs. CD8^+^ T cells were induced by autologous DCs pulsed with HSP70-HER-2-PCs purified from SKBR-3. Cytolytic activities were determined by LDH release assay, as described above, and MDA-MB-231 cells (HER-2 low-expression) were used as controls. The expression of HER-2 in both MDA-MB-453 and MDA-MB-231 cells was previously confirmed by flow cytometry.

### Statistical analysis

Values were expressed as means ± SD or percent (%). All analyses were conducted using the SPSS 13.0 software. The results were considered statistically significant at P<0.05.

## Results

### Identification of HSP70-HER-2-PCs

**Figure 1:**
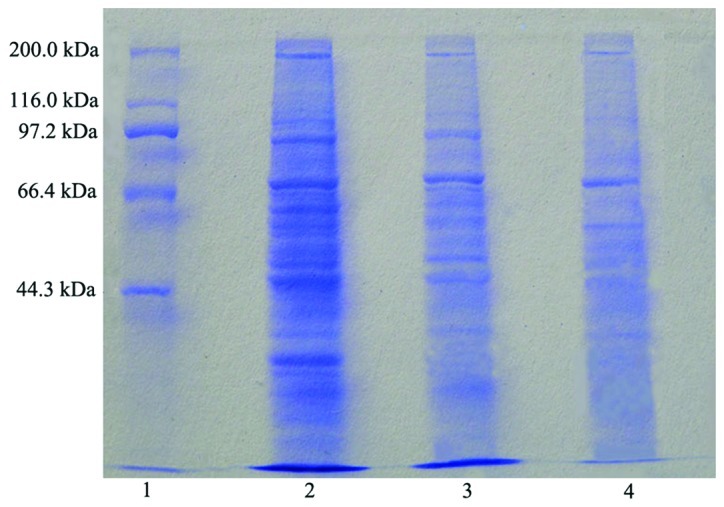
SDS-PAGE analysis for purified fractions. Lane 1, protein molecular marker; lane 2, supernatant after centrifugation; lane 3, unbound parts of the ConA-Sepharose column; lane 4, HSP70-HER-2-PCs.

**Figure 2:**
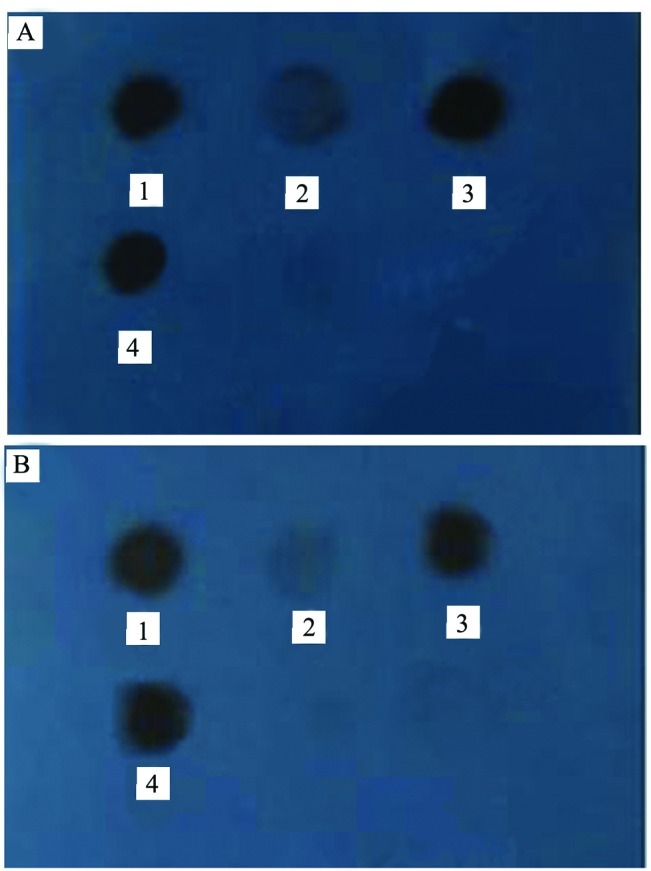
Immunodot analysis for HSP70 and HER-2 protein in purified fractions. (A) Immunodot analysis for HSP70 in purified fractions. Dot 1, supernatant after centrifugation; dot 2, sediments after centrifugation; dot 3, unbound parts of the ConA-Sepharose column; dot 4, HSP70-HER-2-PCs. (B) Immunodot analysis for HER-2 protein in purified fractions. Dot 1, supernatant after centrifugation; dot 2, sediments after centrifugation; dot 3, unbound parts of the ConA-Sepharose column; dot 4, HSP70-HER-2-PCs.

As described in Materials and methods, HSP70-HER-2-PCs were purified from SKBR-3 cells as well as identified by SDS-PAGE with Coomassie Brilliant Blue staining and immunodotting using HSP70-specific and HER-2-specific antibodies. The purified products were found to contain ~70- and 185-kDa proteins (Fig. 1). The purified protein hybridized with the HSP70-specific and HER-2-specific antibodies (Fig. 2A and B), demonstrated that the obtained complex contained HSP70 and HER-2 protein. The other protein straps apart from HSP70 and HER-2 in the result may typify other tumor antigen peptides in the SKBR-3 cells that were bound by HSP70.

Quantitative detection was performed using HER-2 ELISA kits. The content of HER-2 protein in the HSP70-HER-2-PCs was ~2.09 μg. The endotoxin levels in the preparations were lower than 0.03 EU/mg, as determined by an LAL assay.

### Recombinant human HSP70-HER-2 protein complex prepared in vitro

**Figure 3:**
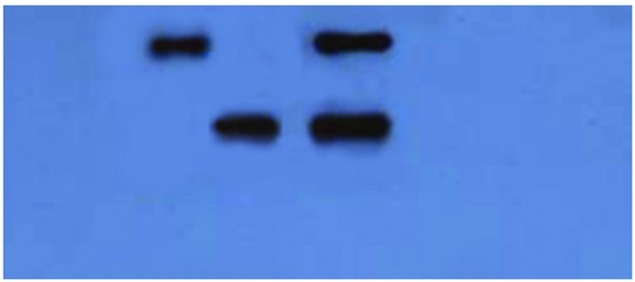
Western blot analysis of the recombinant human HSP70-HER-2 protein complex. Both mouse anti-human monoclonal antibody against HSP70 and rabbit anti-human monoclonal antibody against HER-2 were used in this analysis. Recombinant human HSP70 and HER-2 protein were used as controls.

Recombinant human HSP70 was incubated with recombinant human HER-2 protein at a 1:1 molar ratio at 43°C for 30 min, and then at 37°C for 1 h. The complex was then detected by co-immunoprecipitation and western blot analysis. The protein A-Sepharose-immune complex was found to bind with both mouse anti-human monoclonal antibody against HSP70 and rabbit anti-human monoclonal antibody against HER-2. Using a chemiluminescence reagent, the successful *in vitro* preparation of the recombinant human HSP70-HER-2 protein complex was confirmed (Fig. 3).

### Fluorescence immunostaining

**Figure 4:**
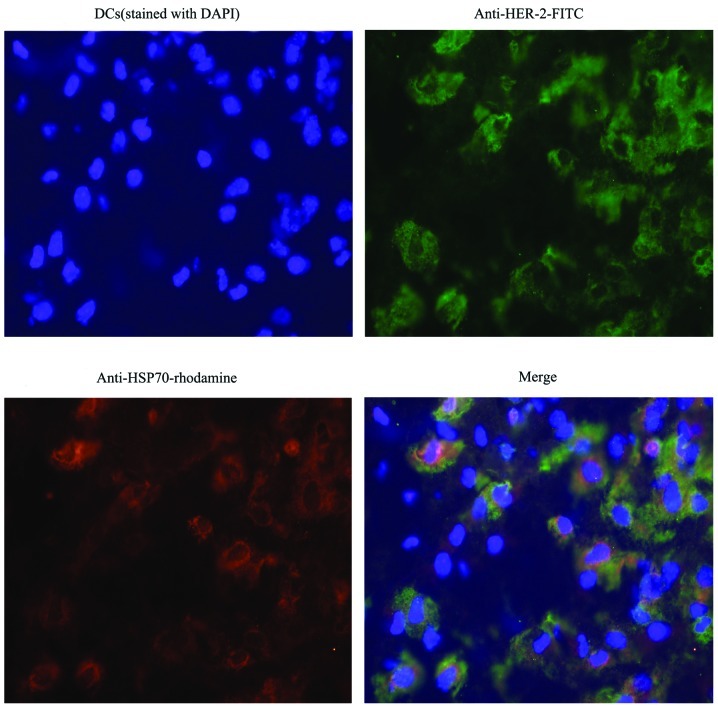
DCs uptake the HSP70-HER-2-PCs. DCs were pulsed by HSP70-HER-2-PCs at 37°C for 12 h. The cells were then incubated with a specific monoclonal antibody against HSP70 or HER-2 protein, and visualized by light microscopy. The surface of the DCs was identified by HSP70 (red color) and HER-2 protein (green color) expression.

The ability of DCs to uptake HSP70-HER-2-PCs purified from SKBR-3 was subsequently determined. DCs were pulsed with HSP70-HER-2-PCs at 37°C for 12 h. After the numerous aforementioned treatments and extensive washing to remove unbound proteins, the DCs were characterized by an Olympus DP71 microscope. Fig. 4 shows that DCs were identified by the presence of HSP70 (red color) and HER-2 protein (green color) expression. Evidently, the HSP70-HER-2-PCs were by taken up by the DCs (Fig. 4).

### Antigen-specific IFN-γ production induced by DCs pulsed with HSP70-HER-2-PCs

**Figure 5:**
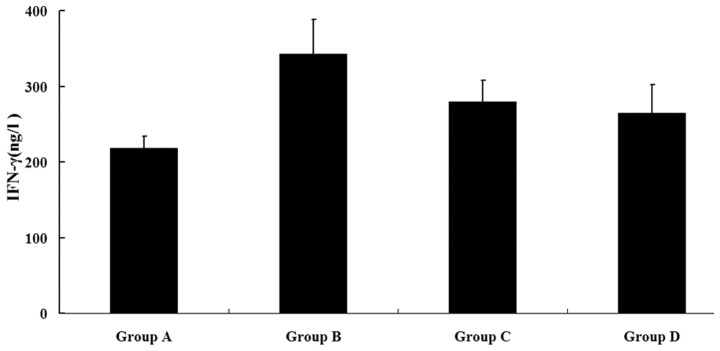
Secretion of IFN-γ by T cells induced with autologous DCs. SKBR-3 cells were the target cells and the effector cells were different among the four groups. Group A includes T cells cocultured with autologous DCs that received only GM-CSF and IL-4. Group B includes T cells cocultured with autologous DCs pulsed with 10 μg of HSP70-HER-2-PCs purified from SKBR-3. Group C includes T cells cocultured with autologous DCs pulsed with 10 μg of HSP70-PCs traditionally purified from SKBR-3. Group D includes T cells cocultured with autologous DCs pulsed with 10 μg of recombinant human HSP70-HER-2 protein complex. Assays were performed in triplicate and the results are expressed as mean ± SD.

Cell-mediated immunity, which is particularly important in tumor suppression, is characterized by the production of type I cytokines. Consequently, the possible ability of DCs pulsed with HSP70-HER-2-PCs to stimulate autologous T cells and induce IFN-γ secretion was explored. Autologous T cells cocultured with the four DC groups were used as effector cells, and SKBR-3 cells were used as target cells. These cells were then detected in the four sample groups using an IFN-γ ELISPOT kit and an ELISA reader system. Fig. 5 shows that the IFN-γ level was significantly higher in group B than in the three other groups (P<0.05). This phenomenon confirmed that the DCs pulsed by HSP70-HER-2-PCs were capable of stimulating autologous T cells and inducing type I cytokine secretion at high levels.

### Induction of specific CD8^+^ T cells by DCs pulsed with HSP70-HER-2-PCs and in vitro cytotoxicity test

**Figure 6:**
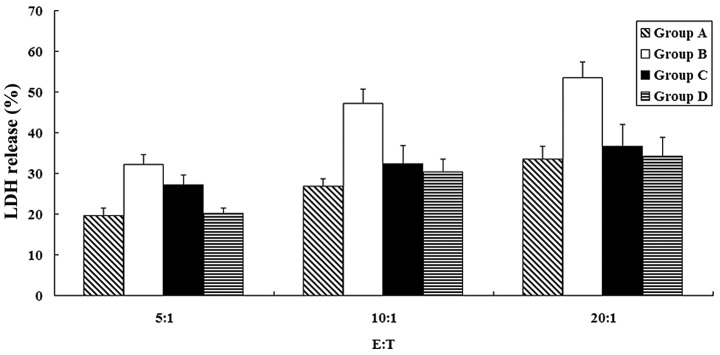
Levels of LDH released by CD8^+^ T cells induced with autologous DCs. SKBR-3 cells were the target cells, and the effector cells were different among the four groups. Group A includes CD8^+^ T cells induced with autologous DCs that received only GM-CSF and IL-4. Group B includes CD8^+^ T cells induced with autologous DCs pulsed with HSP70-HER-2-PCs purified from SKBR-3. Group C represents CD8^+^ T cells induced with autologous DCs pulsed with HSP70-PCs traditionally purified from SKBR-3. Group D includes CD8^+^ T cells induced with autologous DCs pulsed with recombinant human HSP70-HER-2 protein complex. Assays were performed in triplicate and the results are expressed as mean ± SD.

The four groups of CD8^+^ T cells were induced by coculturing with four groups of autologous DCs which were pulsed by different ways (mentioned above). The specific cytolytic activities of CD8^+^ T cells against SKBR-3 cells were next examined by detecting the level of LDH release. This detection was performed after 4 h of coculturing effector cells (CD8^+^ T cells) with target cells (SKBR-3 cells) at different ratios (5:1, 10:1 and 20:1). The LDH release level was found to be significantly higher in group B than in the other groups (P<0.05) (Fig. 6). This finding indicated that the CD8^+^ T cells induced by DCs which were pulsed with HSP70-HER-2-PCs had stronger cytotoxicities against the SKBR-3 cells. Therefore, the HSP70-HER-2-PCs were an improved individual tumor vaccine that had a more effective antigenicity to SKBR-3 cells than other types of HSP70-PCs, and induced more tumor-specific CD8^+^ T cells.

### Inhibition of HER-2 expression by siRNA

**Figure 7:**
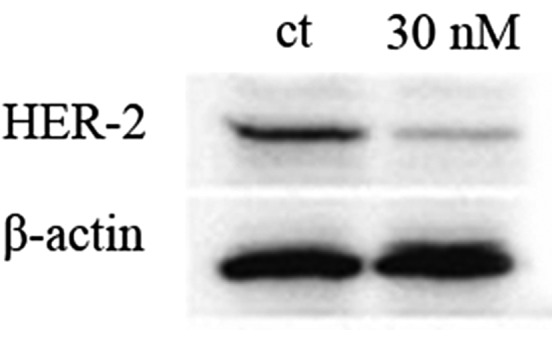
Inhibition of HER-2 expression by siRNA. SKBR-3 cells were transfected with HER-2 siRNA at a concentration of 30 nM, and levels of HER-2 were determined by western blotting. β-actin was used as the internal control.

The knockdown of HER-2 was performed in SKBR-3 cells using siRNA techniques. Expression was evaluated by western blot analysis. The knockdown of HER-2 using the method described above was confirmed to be efficient, as shown in Fig. 7.

### Detection of HER-2 specificity

**Figure 8:**
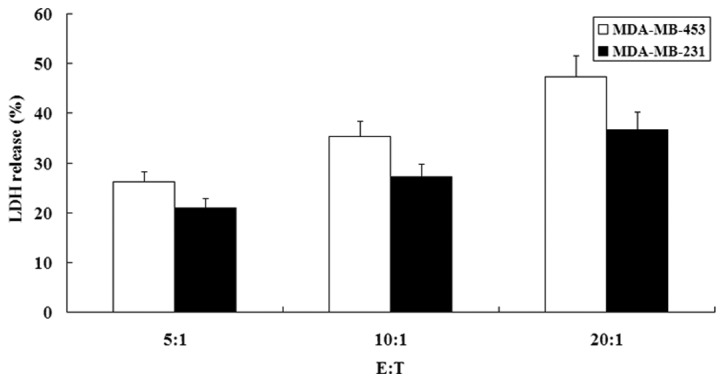
Immunocompetence of HSP70-HER-2-PCs against MDA-MB-453 cells. Effector cells were CD8^+^ T cells induced by autologous DCs pulsed with HSP70-HER-2-PCs purified from SKBR-3. Target cells were MDA-MB-453 and MDA-MB-231 cells. Cytolytic activities of CD8^+^ T cells were determined by LDH release assay, which was performed in triplicate. The results are expressed as mean ± SD.

**Figure 9:**
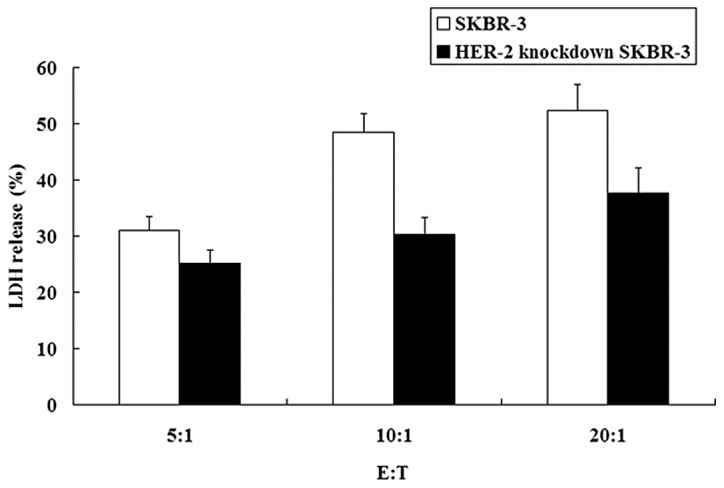
Immunocompetence of HSP70-HER-2-PCs against HER-2-knockdown SKBR-3 cells. Effector cells were CD8^+^ T cells induced by autologous DCs pulsed with HSP70-HER-2-PCs purified from SKBR-3. Target cells were SKBR-3 and HER-2-knockdown SKBR-3 cells. Cytolytic activities of CD8^+^ T cells were determined by LDH release assay, which was performed in triplicate. The results are expressed as mean ± SD.

HER-2 specificity with HSP70-HER-2-PCs will determine the application scope of the immune complex. We confirmed the characteristics of the complex using two trials. First, the cytotoxicity of effector cells against MDA-MB-453 and MDA-MB-231 cells were compared. Effector cells showed significant cytotoxicity against MDA-MB-453 cells, but exhibited minimal lysis against MDA-MB-231 cells (P<0.05) (Fig. 8). Moreover, the cytotoxicities of effector cells against SKBR-3 and HER-2-knockdown SKBR-3 cells were compared. Effector cells showed stronger cytotoxicity against SKBR-3 cells than HER-2-knockdown SKBR-3 cells (P<0.05) (Fig. 9).

## Discussion

HSP70-PCs purified from tumor cells elicit anticancer immunity when used as a tumor vaccine (17). The immune response stimulated by HSP70-PCs is considered to consist of two parts. First is the delivery of antigens for cross-presentation on the MHC class I molecules of DCs. Second is the stimulation of DCs to excrete cytokines and express costimulatory molecules, thereby creating the immunogenic environment required for the induction of CD8^+^ T cell responses (18). Previous studies have shown that immunization of mice with HSP70-PCs provides protection against tumor cells from which the HSP70-PCs were purified or slows the progression of those tumors (19,20). Based on these results, HSP70-based vaccines are purified from the resected tumors of patients to whom the vaccine will be applied. Therefore, HSP70 in itself has no antigenicity, and its immunogenicity is attributed to the peptides it chaperones or binds with (8,11,21,22). The content and kind of antigenic peptides in HSP70-PCs that are isolated from tumor cells determine the immune response against the same tumor. Consequently, research on HSP70-based tumor immunotherapy is focused on how to obtain more effective and powerful peptides by the purification of HSP70-PCs from tumor cells.

Previously purified HSP70-PC-based vaccines, while eliciting immunological responses, have been proven to be insufficient in providing protection against tumor growth. Thus, the efficacy of HSP70-PCs as a therapeutic tumor vaccine requires further improvement. To date, no evidence showing that membrane tumor-associated peptides, such as HER-2 protein, are produced by the purification of HSP70-PCs from tumor cells is available. Since older HSP70-based purification methods cannot completely separate the cellular membrane, losses in membrane antigenic peptides are often observed.

In this study, HER-2 protein was used to represent membrane antigens to verify the new product. HER-2 is an important and ideal tumor antigen peptide that is mostly expressed on the cell membrane. It is highly relevant to breast cancer and other cancers (e.g. ovarian, prostate, lung, and colon). Some patients with cancer, especially that of the breast, have a pre-existing immune responses directed against HER-2 (23,24). Therefore, an effective cancer vaccine targeting HER-2 protein will be able to boost this immunity to therapeutic levels (25). In addition, HER-2 protein can be easily identified.

Some researchers have used recombinant *in vitro* heat shock-bound HSP-HER-2 protein complexes as cancer vaccines for HER-2 target treatment. They confirmed that recombinant HSP-HER-2 protein complexes elicit an antigen-specific CD8^+^ T cell response. Nevertheless, this immune response is insufficient since there are various types of important antigenic peptides in tumor cells besides HER-2 protein. Although the concrete identity of these peptides has yet to be identified, they still contribute to the antitumor immunocompetence of HSP70-based vaccines. The recombinant human HSP70-HER-2 protein complex was successfully prepared *in vitro* and used as an important control to support this theory (Fig. 3).

The present study extended previous findings and established a new method using CHAPS to purify HSP70-PCs containing membrane-associated tumor peptides from human cancer cells. CHAPS was chosen because it is suitable for laminar analyses and easily depleted by dialysis.

The product, HSP70-HER-2-PCs containing both membrane-resident tumor peptides, HER-2 protein as a representative, and other antigenic peptides bound by HSP70, were able to induce tumor-specific CD8^+^ T cells with higher specificity against SKBR-3 cells than traditional HSP70-PCs and recombinant human HSP70-HER-2 protein complexes (Figs. 5 and 6). Moreover, HSP70-HER-2-PCs possessed HER-2 specificity and could be used as a HER-2 target vaccine for treating other HER-2 overexpressed tumor cells (Figs. 8 and 9).

In summary, an improved method for preparing an advanced HSP70-based vaccine from cancer cells was designed. This approach was based on HSP70 and a purification process involving the detergent CHAPS to obtain membrane-resident tumor-associated peptides from cancer cells. The product exhibited stronger immunogenic activity and served as a better tumor antigen source for pulsing DCs. The pulsed DCs were able to induce more effective tumor-specific CD8^+^ T cells. Animal experiments are ongoing by our group to determine the immunocompetence of the new HSP70-based vaccine *in vivo*. The findings of this study provide the basis for a new approach for enhancing HSP70-based immunotherapies for HER-2-associated or other membrane antigenic peptide-related tumors. We firmly believe that the new customized vaccine will provide tumor patients with better individualized treatments.

## References

[1] Hightower LE (1991). Heat shock, stress proteins, chaperones, and proteotoxicity. Cell.

[2] Pandey M, Mathew A, Nair MK (1999). Cancer vaccines: a step towards prevention and treatment of cancer. Eur J Surg Oncol.

[3] Hartl FU (1996). Molecular chaperones in cellular protein folding. Nature.

[4] Srivastava PK (2005). Immunotherapy for human cancer using heat shock protein-peptide complexes. Curr Oncol Rep.

[5] Srivastava PK (1993). Peptide-binding heat shock proteins in the endoplasmic reticulum: role in immune response to cancer and in antigen presentation. Adv Cancer Res.

[6] Sherman M, Multhoff G (2007). Heat shock proteins in cancer. Ann NY Acad Sci.

[7] Huang C, Yu H, Wang Q (2004). Potent antitumor effect elicited by superantigen-linked tumor cells transduced with heat shock protein 70 gene. Cancer Sci.

[8] Srivastava PK, DeLeo AB, Old LJ (1986). Tumor rejection antigens of chemically induced sarcomas of inbred mice. Proc Natl Acad Sci USA.

[9] Udono H, Levey DL, Srivastava PK (1994). Cellular requirements for tumor-specific immunity elicited by heat shock proteins: tumor rejection antigen gp96 primes CD8^+^ T cells in vivo. Proc Natl Acad Sci USA.

[10] Tamura Y, Peng P, Liu K, Daou M, Srivastava PK (1997). Immunotherapy of tumors with autologous tumor-derived heat shock protein preparations. Science.

[11] Vanaja DK, Grossmann ME, Celis E, Young CY (2000). Tumor prevention and antitumor immunity with heat shock protein 70 induced by 15-deoxy-delta12, 14-prostaglandin J2 in transgenic adenocarcinoma of mouse prostate cells. Cancer Res.

[12] Suto R, Srivastava PK (1995). A mechanism for the specific immunogenicity of heat shock protein-chaperoned peptides. Science.

[13] Noessner E, Gastpar R, Milani V (2002). Tumor-derived heat shock protein70 peptide complexes are cross-presented by human dendritic cells. J Immunol.

[14] Kumar S, Deepak P, Acharya A (2009). Autologous HSP70 immunization induces anti-tumor immunity and increases longevity and survival of tumor-bearing mice. Neoplasma.

[15] Manjili MH, Henderson R, Wang XY (2002). Development of a recombinant HSP110-HER-2/neu vaccine using the chaperoning properties of HSP110. Cancer Res.

[16] Thurner B, Roder C, Dieckmann D (1999). Generation of large numbers of fully mature and stable dendritic cells from leukapheresis products for clinical application. J Immunol Methods.

[17] Udono H, Srivastava PK (1993). Heat shock protein 70-associated peptides elicit specific cancer immunity. J Exp Med.

[18] Bendz H, Ruhland SC, Pandya MJ (2007). Human heat shock protein 70 enhances tumor antigen presentation through complex formation and intracellular antigen delivery without innate immune signaling. J Biol Chem.

[19] Ciupitu AM, Petersson M, Kono K, Charo J, Kiessling R (2002). Immunization with heat shock protein 70 from methylcholanthrene-induced sarcomas induces tumor protection correlating with in vitro T cell responses. Cancer Immunol Immunother.

[20] Chen DX, Su YR, Shao GZ, Qian ZC (2004). Purification of heat shock protein 70-associated tumor peptides and their antitumor immunity to hepatoma in mice. World J Gastroenterol.

[21] Palladino MA, Srivastatva PK, Oettgen HF, DeLeo AB (1987). Expression of a shared tumor-specific antigen by two chemically induced BALB/c sarcomas. Cancer Res.

[22] Srivastava PK, Udono H (1994). Heat shock protein-peptide complexes in cancer immunotherapy. Curr Opin Immunol.

[23] Disis ML, Cheever MA (1997). HER-2/neu protein: a target for antigen-specific immunotherapy of human cancer. Adv Cancer Res.

[24] Disis ML, Grabstein KH, Sleath PR, Cheever MA (1999). Generation of immunity to the HER-2/neu oncogenic protein in patients with breast and ovarian cancer using a peptide-based vaccine. Clin Cancer Res.

[25] Lin KY, Guarnieri FG, Staveley-O’Carroll KF, Levitsky HI, August JT, Pardoll DM, Wu TC (1999). Treatment of established tumors with a novel vaccine that enhances major histocompatibility class II presentation of tumor antigen. Cancer Res.

